# TSLC: differential abundance analysis method for identifying biologically relevant known microbes

**DOI:** 10.1093/bioadv/vbag297

**Published:** 2026-10-06

**Authors:** Robert Beblavy, Cynthia L Monaco, Cassandra E Newkirk, Jin Xiao, Michael B Sohn

**Affiliations:** Biostatistics and Computational Biology, University of Rochester Medical Center, Rochester, NY 14642, United States; Microbiology and Immunology, University of Rochester Medical Center, Rochester, NY 14642, United States; Medicine, University of Rochester Medical Center, NY 14642, United States; Microbiology and Immunology, University of Rochester Medical Center, Rochester, NY 14642, United States; Eastman Institute for Oral Health, University of Rochester Medical Center, Rochester, NY 14620, United States; Biostatistics and Computational Biology, University of Rochester Medical Center, Rochester, NY 14642, United States

## Abstract

**Motivation:**

Identifying biologically relevant microbes is a crucial task in human microbiome studies. Numerous computational and statistical methods have been proposed for this task. However, most existing methods are based solely on taxonomic information, even when whole-metagenome sequencing data are available, and thus cannot account for the fact that different microbes can perform the same or similar functions (known as *functional redundancy*) and for the contribution of unknown microbes to functional abundance. Hence, the identified differentially abundant microbes might not be biologically relevant to manifested phenotypes.

**Results:**

To address the issues with existing methods, we propose a two-stage procedure: (i) determining differentially abundant functions between groups; and then (ii) determining microbes significantly associated with each identified differentially abundant function. This procedure involves $\ell_{1}$-penalized log-contrast models and debiasing procedures to account for the compositional nature of microbiome data and the bias induced by the $\ell_{1}$ penalty. We performed extensive simulation studies to illustrate the consequences of ignoring functional redundancy and the contribution of unknown microbes, and to assess the performance of the proposed method. We applied the proposed method to two whole-metagenome sequencing datasets and showcased substantial differences between the typical taxonomic-based analysis and the proposed method.

**Availability and implementation:**

An R package for the proposed method is available at https://github.com/rgbeblavy/TSLC.

## 1 Introduction

The human microbiome is the collection of all microorganisms residing in the human body and has been shown to play a vital role in maintaining health (Li 2015). A common objective in microbiome data analysis is differential abundance (DA) analysis, which aims to identify microbes whose abundances differ significantly between groups. Microbiome data are, by nature, compositional as they consist of relative information (Gloor *et al.* 2017). However, classical statistical methods that do not account for the compositional nature of the data have often been used for DA analysis in microbiome studies (Hawinkel *et al.* 2017). The use of such methods can thus lead to spurious results (Aitchison 1986), as the abundance of a taxon cannot change without affecting the abundance of other taxa due to the unit-sum constraint. Moreover, microbiome data are high-dimensional, which makes DA analysis more challenging.

Some DA analysis methods, such as an $\ell_{1}$-penalized log-contrast model (LCM) (Lu *et al.* 2019), address these challenges. However, these methods examine only which microbes differ in abundance between groups and do not consider their potential functions. Therefore, the identified DA microbes may not be relevant to the different phenotypes between groups. This can be a problem, as translating microbiome research into therapeutic applications requires identifying DA microbes that are relevant to distinct phenotypes across groups. From a biological perspective, we are interested only in those DA microbes that exhibit differences in observable characteristics. Throughout this article, we will refer to these microbes as biologically relevant microbes to phenotype and denote them as BR_p_ microbes. Note that DA microbes just need to be different in their abundance across groups, while BR_p_ microbes must be functionally different, potentially or actually, across groups.

It is known that functional profiles tend to be quite similar across individuals, although taxonomic profiles can be different across individuals, indicating functional redundancy (i.e. different groups of microbes can perform the same function) (Manor and Borenstein 2017, Gilbert *et al.* 2018, Louca *et al.* 2018, Tian *et al.* 2020, Puente-Sánchez *et al.* 2024). The consequence of functional redundancy on DA analysis, as well as the contributions of known microbes and unknown microbes (i.e. not assigned to or not in a reference database) to functional shifts, is illustrated in Fig. 1, which depicts five possible cases in a two-group comparison. The top bar represents the abundance of a function in one group (e.g. healthy) contributed by many known and unknown microbes, while the bottom bar represents that in another group (e.g. diseased). Here, a function can represent a gene family or a pathway.

**Figure 1:**
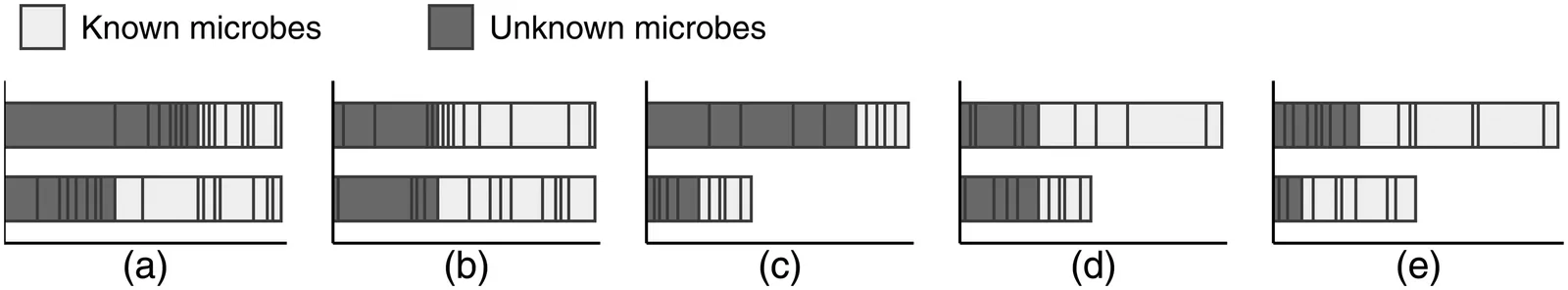
Possible cases for the contributions of known and unknown microbes in the functional composition for two-group comparison. The upper bar represents one group, and the lower bar another group.

Cases (a) and (b) illustrate functional redundancy, and they differ in the overall contributions of known and unknown microbes. For Case (a), disregarding the contribution of unknown microbes will result in the erroneous identification of the function as differentially abundant, leading to incorrect conclusions regarding the association of related taxa with phenotypic differences between groups. This highlights the need to consider the contributions of unknown microbes to functional shifts. It is the aggregated contribution of all unknown microbes, rather than individual unknown taxa, that is relevant when identifying BR_p_ microbes. The remaining cases depict the overall contributions of known and unknown microbes to differentially abundant functions, with the known microbes being potential BR_p_ microbes we are trying to identify. Although the total contribution of known taxa in Case (c) is the same across the two groups, the abundance of individual microbes may still vary, so they may still be BR_p_ microbes.

To the best of our knowledge, the only method that has attempted to connect the taxonomic and functional profiles to find BR_p_ known taxa is FishTaco (Manor and Borenstein 2017). FishTaco requires two input datasets and one optional input dataset: (i) a taxonomic profile *M*, (ii) a functional profile *F*, and optionally (iii) a matrix of gene copy numbers *G*. In the first stage, FishTaco identifies DA functions between groups using a Wilcoxon test. In the second stage, it quantifies each taxon’s contribution to the observed functional differences by repeatedly permuting taxon abundances and measuring the resulting changes in the function. The permutation procedure breaks the association between an individual taxon and the outcome, enabling FishTaco to determine how much the taxon contributes to the outcome. For each permutation, it obtains the marginal contribution of each microbe for all functions using a Wilcoxon test. Finally, it uses Shapley value analysis to quantify the contributions of individual taxa to the functions.

Although FishTaco provides a framework for identifying BR_p_ known taxa, it does not properly account for the compositional nature of the data, as it assesses significance based directly on relative abundance. Because of the unit-sum constraint on the taxonomic and function profiles, a change in the abundance of one taxon (or function) will necessarily result in changes in the abundance of other taxa (or functions), which induces an artificial effect, known as the compositional effect. As a result of ignoring this compositional effect, the BR_p_ known taxa identified by FishTaco may be due to the compositional effect rather than actual changes in abundance. Moreover, it does not explicitly consider unknown species in its model. This motivates us to develop a framework that accounts for the compositional nature of the microbiome data and explicitly considers unknown species in identifying BR_p_ known taxa.

To address the compositionality and the contribution of unknown microbes, we propose a method that takes a two-step procedure: (i) identify DA functions and (ii) find which known microbes are significantly associated with these DA functions, i.e. find BR_p_ microbes. At each step, we use an $\ell_{1}$-penalized LCM to account for compositionality, and a debiasing procedure to correct bias in the estimated regression coefficients induced by the $\ell_{1}$ penalty under the sparsity assumption. We demonstrate, through simulation studies, that the proposed method consistently identifies BR_p_ microbes, whereas competing DA methods do not. In real data analysis, we show a consequence of ignoring metagenome-based functional redundancy and the contribution of unknown microbes to the identification of known microbes associated with observed phenotypes.

## 2 Methods

### 2.1 Two-stage procedure to find BR_p_ microbes

To describe how the known and unknown microbes are linked to functions, let us denote by *M* an n×k matrix containing abundance levels of microbes measured from the whole-metagenome sequencing data, by *G* a k×p matrix containing genomic content of *k* microbes extracted from a reference metagenome database, and by *F* an n×p matrix containing abundance levels of functions measured from the whole-metagenome or metatranscriptome sequencing data, where *n* is the number of samples, *p* is the number of functions, and *k* is the number of microbes consisting of $k^{\prime }$ known microbes and $k-k^{\prime }$ unknown microbes. Then, a functional profile can be equated with genomic content weighted by the abundance levels of microbes:


(1) $$F=MG=M_{\text{known}}G_{\text{known}}+M_{\text{unknown}}G_{\text{unknown}},$$

where $M_{\text{known}}$ is an $n\times k^{\prime }$ abundance matrix of known microbes, $M_{\text{unknown}}$ is an $n\times (k-k^{\prime })$ abundance matrix of unknown microbes, and $G_{\text{known}}$ and $G_{\text{unknown}}$ are similarly defined. Note that $M_{\text{unknown}}$ and $G_{\text{unknown}}$ are not separately identifiable. However, their product, which measures the extent to which all unknown microbes contribute to functional shifts, is identifiable. This overall contribution from unknown microbes, or the abundance of sequencing reads assigned to the unknown or unclassified taxa, $MG_{\text{unknown}}$, is sufficient to estimate the contribution of known microbes to functional shifts.

Identifying BP_p_ microbes requires a functional profile *F* and the contribution of taxa to functional shifts, i.e. $M_{\left(j\right)}=M⊙(1⊗g_{j}^{⊤})$ for function *j*, where ⊙ represents the Hadamard product, 1 a vector of 1s, ⊗ the outer product, and $g_{j}$ the *j*th column of *G*. These can be obtained directly from a functional profiling method, such as HUMAnN (Beghini *et al.* 2021), which profiles the abundance of microbial gene families or pathways *F* from whole-metagenome or metatranscriptome sequencing data and the contributions of known and unknown microbes to each gene family or pathway $M_{\left(j\right)}$ for j=1,2,…,p. $M_{\left(j\right)}$ reflects the contribution of taxa to the *j*th potential function when *F* is measured from whole-metagenome sequencing data, whereas it reflects the contribution of taxa to the *j*th expressed function when *F* is measured from metatranscriptome sequencing data. Note that *F* is compositional data and $M_{\left(j\right)}$ is subcompositional data, which can be recasted as compositional data, as a result of transforming counts into proportions or rarefying counts.

#### 2.1.1 Stage 1: determining DA functions

To identify DA functions given a function profile *F*, we solve the following optimization problem of an $\ell_{1}$ penalized probit LCM:


(2) $$\begin{array}{cc} \hat{\alpha } & =\underset{\alpha }{\text{argmin}}\{-\frac{1}{n}\sum_{i=1}^{n}L[y_{i}|\;\text{log}(f_{i\cdot }),\alpha ]+\lambda \sum_{j=1}^{p}|\alpha_{j}|\},\\ & \;\text{subject}\;\text{to}\;\sum_{j=1}^{p}\alpha_{j}=0, \end{array}$$

where $y_{i}$ is the group membership, L(·) is the likelihood function for probit model, $f_{i\cdot }$ is the *i*th row vector of *F*, and λ≥0 is a penalty parameter. Note that penalization of the regression coefficients is necessary, since the number of functions is typically the same order of magnitude as, or larger than, the number of samples. The zero-sum constraint of the coefficient is crucial, as $f_{i\cdot }$ is compositional (Aitchison and Bacon-Shone 1984, Lin *et al.* 2014).

To solve Eq. (2) computationally efficiently, we use the Taylor expansion of the log-likelihood function for the probit model. Specifically, we first transform $y_{i}$ such that it takes on {−1,+1} and define $X=[1,\;\text{log}(\tilde{F})]$, where $\text{log}(\tilde{F})=\text{log}(F)(I_{p}-J_{p}/p)$, where $I_{p}$ is the p×p identity matrix and $J_{p}$ is the p×p matrix of ones. Note that $\text{log}(F)\alpha_{-0}=\text{log}(\tilde{F})\alpha_{-0}$ because of the zero-sum constraint, where $\alpha_{-0}$ indicates the exclusion of the coefficient for the intercept, so this transformation will not affect the estimates of α. It is necessary for a debiasing procedure, described subsequently in this section. Then, using the Taylor expansion, we express the optimization problem Eq. (2) as


(3) $$\begin{array}{cc} \hat{\alpha } & =\underset{\alpha }{\text{argmin}}\{\frac{1}{2n}||D^{1/2}(u-X\alpha )|{|}_{2}^{2}+\lambda \sum_{j=1}^{p}|\alpha_{j}|\},\\ & \;\text{subject}\;\text{to}\;\sum_{j=1}^{p}\alpha_{j}=0, \end{array}$$

where *D* is an n×n diagonal matrix with its *i*th diagonal term $D_{ii}=\xi_{i}(\tilde{\alpha })[x_{i\cdot }^{⊤}\tilde{\alpha }+\xi_{i}(\tilde{\alpha })]$, where $\tilde{\alpha }$ is a vector lying between a neighborhood $\underline{\alpha }$ in the Taylor expansion and $\hat{\alpha }$, $x_{i\cdot }$ is the *i*th row vector of *X*, and $\xi_{i}(\alpha )=y_{i}\phi (y_{i}x_{i\cdot }^{⊤}\alpha )/\Phi (y_{i}x_{i\cdot }^{⊤}\alpha )$, where ϕ(·) and Φ(·) are the probability density function and cumulative density function of the standard normal distribution, respectively, and $u=X\underline{\alpha }+D^{-}\xi (\underline{\alpha })$, where $D^{-}$ is the Moore–Penrose inverse of *D* and $\xi (\underline{\alpha })=(\xi_{1}(\underline{\alpha }),\ldots ,\xi_{n}(\underline{\alpha }))$. The details of transforming Eq. (2) into Eq. (3) using a Taylor expansion are given in the Supplementary File.

This alternative optimization problem has a form of weighted least squares, but *D* and u depend on $\tilde{\alpha }$ and α, respectively. Thus, we solve the optimization problem by combining iteratively reweighted least squares (IRLS) and the coordinate descent method of multipliers (CDMM). This method involves constructing the augmented Lagrangian and iteratively updating each of these quantities. The augmented Lagrangian (Bertsekas 2009) for the optimization problem [Eq. (3)] is given by


$$L=\frac{1}{2n}||D^{1/2}(u-X\alpha )|{|}_{2}^{2}+\lambda \sum_{j=1}^{p}|\alpha_{j}|+\frac{\omega }{2}{\left(\sum_{j=1}^{p}\alpha_{j}+\eta \right)}^{2},$$

where η is a scaled Lagrange multiplier and ω>0 is a penalty parameter. The coefficients α, as the $\ell_{1}$ penalty term is separable, can be updated using the coordinate descent method:


(4) $$\alpha_{j}^{\left(k+1\right)}\leftarrow \frac{1}{m_{j}}S_{\lambda }\{h(X,\alpha_{-j})-\omega g(\alpha_{-j},\eta )\},$$

where $m_{j}=||x_{\cdot j}|{|}_{2}^{2}/n+\omega /p$,


$$\begin{array}{c} h(X,\alpha_{-j})=\frac{1}{n}x_{\cdot j}^{⊤}D^{\left(k\right)}(u^{\left(k\right)}-X_{-j}\alpha_{-j}),\\ g(\alpha_{-j},\eta )=\frac{1}{p}\sum_{\ell \neq j}\alpha_{\ell }^{\left(k+1\right)}+\frac{1}{\sqrt{p}}\eta^{\left(k\right)}, \end{array}$$

and $S_{\lambda }(t)=\text{sign}(t){\left(|t|-\lambda \right)}_{+}$ is the soft thresholding operator. The scaled Lagrange multiplier η can then be updated using a gradient descent method:


(5) $$\eta^{\left(k+1\right)}\leftarrow \eta^{\left(k\right)}+\sum_{j=1}^{p}\alpha_{j}^{\left(k+1\right)}.$$

The other terms *D* and u at iteration k+1 can be updated with $\alpha^{\left(k+1\right)}$, $\eta^{\left(k+1\right)}$, and ${\tilde{\alpha }}^{\left(k+1\right)}$ that can be estimated by a line search that maximizes $\sum_{i=1}^{n}\;\text{log}\;\Phi (y_{i}x_{i\cdot }^{⊤}\alpha^{\left(k+1\right)})$.

To test which functions are differentially abundant between two groups, we apply a debiasing procedure to correct the bias in $\hat{\alpha }$ due to the $\ell_{1}$ penalty. Specifically, we utilize the KKT condition and construct the debiased estimator of $\hat{\alpha }$ as


$${\hat{\alpha }}^{\text{de}}=\hat{\alpha }+\frac{1}{n}\tilde{\Theta }X^{⊤}\hat{D}(\hat{u}-X\hat{\alpha }),$$

where $\tilde{\Theta }=(I_{p}-J_{p}/p)\hat{\Theta }$ and $\hat{\Theta }$ is an approximate inverse matrix of $\hat{\Sigma }=X^{⊤}\hat{D}X/n$. Under some regularity conditions (Sohn *et al.* 2021), it can be shown that


$$\sqrt{n}({\hat{\alpha }}^{\text{de}}-\alpha )\overset{d}{\to }N(0,\sigma^{2}\tilde{\Theta }\hat{\Sigma }{\tilde{\Theta }}^{⊤}).$$

Therefore, we can make an inference on the debiased estimator ${\hat{\alpha }}^{\text{de}}$. To obtain $\hat{\Theta }={\left({\hat{\theta }}_{1},\ldots ,{\hat{\theta }}_{p}\right)}^{⊤}$, we solve the following optimization problem,


(6) $$\begin{array}{cc} & {\hat{\theta }}_{j}=\underset{\theta_{j}}{\text{argmin}}\theta_{j}^{⊤}\hat{\Sigma }\theta_{j},\\ \text{subject}\;\text{to} & ||\hat{\Sigma }\theta_{j}-(I_{p}-J_{p}/p)e_{j}|{|}_{\infty }\leq \gamma , \end{array}$$

where $e_{j}$ is the *j*th canonical basis vector and γ is some constant. The algorithm for Eq. (6) is given in Supplementary File. At the end of this stage, we have a set of DA functions, $S_{F}=\{j\;|\;\alpha_{j}\neq 0,\;j=1,\ldots ,p\}$.

**Algorithm 1** TSLC 1: **Stage 1: Identify DA functions** 

2: Initialize $\alpha^{\left(0\right)}$, $\eta^{\left(0\right)}$, $D^{\left(0\right)}$, $u^{\left(0\right)}$

3: **repeat** 

4:   **for**  j=1 to *p* **do** 

5:     Update $\alpha_{j}^{\left(k+1\right)}$ using (4)

6:     Update $\eta^{\left(k+1\right)}$ using (5)

7:   **end for** 

8:   Find ${\tilde{\alpha }}^{\left(k+1\right)}$ that maximizes $\sum_{i=1}^{n}\;\text{log}\;\Phi (y_{i}x_{i}^{⊤}\alpha )$

9:   Update $D^{\left(k+1\right)}$ and $u^{\left(k+1\right)}$

10:  k←k+1

11: **until** convergence

12: **Stage 2: Identify BR_p_ microbes in each DA function** 

13: **for**  $j_{s}$  ∈  $S_{F}$  **do** 

14:   Initialize $\alpha_{j_{s}}^{\left(0\right)}$, $\eta^{\left(0\right)}$

15:   **repeat** 

16:     **for**  ν=1 to $k_{j_{s}}$  **do** 

17:       Update $\alpha_{\nu }^{\left(k+1\right)}$

18:       Update $\eta^{\left(k+1\right)}$

19:     **end for** 

20:     k←k+1

21:   **until** convergence

22: **end for** 

#### 2.1.2 Stage 2: determining BR_p_ microbes in each DA function

In this stage, we identify BR_p_ known microbes for each of the DA functions determined in Stage 1. Let $j_{s}$ indicate the *s*th function in the set of DA functions $S_{F}$ and $k_{j_{s}}$ be the number of microbes related to the *s*th function. Given $f_{j_{s}}$ (the $j_{s}$th column vector of *F*) and $M_{\left(j_{s}\right)}$ for each $j_{s}\in S_{F}$, we separately solve the following $\ell_{1}$ penalized LCM (Lin *et al.* 2014, Shi *et al.* 2016):


$$\begin{array}{cc} {\hat{\alpha }}_{j_{s}} & =\underset{\alpha }{\text{argmin}}\{\frac{1}{2n}||\;\text{log}(f_{j_{s}})-\text{log}(M_{\left(j_{s}\right)})\alpha_{j_{s}}|{|}_{2}^{2}+\lambda_{j_{s}}\sum_{\nu =1}^{k_{j_{s}}}|\alpha_{j_{s}\nu }|\},\\ & \;\text{subject}\;\text{to}\;\sum_{\nu =1}^{k_{j_{s}}}\alpha_{j_{s}\nu }=0, \end{array}$$

where $\lambda_{j_{s}}\geq 0$ is a penalty parameter for the $j_{s}$th selected function. Note that since the contributions of individual unknown microbes to a functional shift are not identifiable, nor of interest, all unknown microbes are treated as an unknown artificial microbe in the estimation of their contributions to the functional shift. The end result of this stage consists of BR_p_ known microbes for each DA function.

At each stage, we used the Benjamini–Hochberg procedure for multiple testing correction (Benjamini and Hochberg 1995). The algorithm for our two-stage approach is given in Algorithm 1.

### 2.2 Simulation studies

To assess the proposed method, we simulated taxonomic profiles using negative binomial (NB) and logistic-normal (LN) models, and constructed functional profiles using Eq. (1). For the NB model, we used its probability mass function with the following parameterization:


$$P(M=m)=\frac{\Gamma (m+\phi )}{\Gamma (\phi )\Gamma (m+1)}{\left(\frac{\phi }{\phi +\mu }\right)}^{\phi }{\left(\frac{\mu }{\phi +\mu }\right)}^{m},$$

thus, E(M)=μ and $\text{Var}(M)=\mu +\mu^{2}/\phi$, where μ is a mean count and ϕ is a dispersion parameter. For the LN model, we modeled a taxonomic profile using algebraic operators in a simplex space:


$$m_{i\cdot }=m_{0}⊕a^{y_{i}}⊕h^{x_{i}}⊕u_{i\cdot },$$

where $m_{0}$ is a baseline composition, $y_{i}$ is a group indicator, a is a group effect coefficient, $x_{i}$ is a covariate, h is a covariate effect coefficient, and $u_{i\cdot }$ is an LN disturbance variable, i.e. $u_{i\cdot }∼\text{LN}(0,\sigma^{2}N)$, where $N=I_{k-1}+J_{k-1}$. The expressions of the algebraic operators in a simplex space are given in the Supplementary File. Note that the count of each microbe in the NB model is generated independently from other microbes, whereas the abundance levels of all microbes in the LN model are jointly generated and correlated. In performance comparison, we included a two-stage Wilcoxon rank sum test (TSWRS), FishTaco, ANCOM-BC (Lin and Peddada 2020), LinDA (Zhou *et al.* 2022), and one-stage versions (i.e. taxonomic-based DA analysis) of the Wilcoxon rank sum test (WRS) and an $\ell_{1}$-penalized LCM. To make a direct comparison between the one-stage and the two-stage methods, we used the same amount of genomic content for all microbes.

### 2.3 Data generation

The mean count $\mu_{j}$ of each microbe was randomly generated from {1,2,…,20}. These mean counts were then used to simulate the taxonomic profile *M* from the NB or LN models. We added a pseudocount of 0.5 to all zero values and converted counts into proportions. We then constructed the genomic content matrix *G* to indicate which microbes are associated with each function. A microbe is associated with a particular function if it has a nonzero value in the genomic content matrix, and it is not associated with the function if it has a zero value in the genomic content matrix. Using these matrices, the functional profile was constructed from log(F)=log(M)G+ϵ, where $\epsilon ∼N(0,0.5I_{n})$. The group membership y was generated randomly using a Bernoulli distribution with the success probability determined by logistic regression. In particular, let α be the regression coefficients for a logistic regression of group membership on log(F). We set $\alpha_{1},\ldots ,\alpha_{6}\neq 0$ to ensure that the first six functions are differentially abundant. Then, for each subject *i*, the group membership $y_{i}\in \{0,1\}$ is generated from Bernoulli distribution with success probability ${\left(1+\text{exp}(\text{log}\;{\left(f_{i\cdot }\right)}^{⊤}\alpha )\right)}^{-1}$.

Moreover, with a real dataset from Franzosa *et al.* (2019), we generated data under null settings (i.e. all functions and microbes are not differentially abundant) by permuting the group membership $y_{i}$, while keeping the functional profile, taxonomic profile, and covariates the same, to assess the false positive rates of the existing methods and the proposed method.

### 2.4 Microbial profiles by NB models

We considered the case where the taxonomic profile is generated using the NB model with n=50,75 per group, p=80 functions, and k=100 microbes. All results are based on 200 repetitions. As our primary interest is to identify microbes with the potential for functional shift, we consider only BR_p_ microbes as true positives when assessing method performance. In other words, a microbe must be associated with a DA function to be considered a true positive. Figure 2a shows performance comparison results for the first stage, where we identify DA functions, and Fig. 2b shows performance comparison results for the second stage, where we identify BR_p_ microbes when n=50. Figure 2c and d shows performance comparison results when n=75. The proposed method (TSLC) substantially outperformed both FishTaco and TSWRS in identifying DA functions in the first stage and BR_p_ microbes in the second stage. The performance of FishTaco and TSWRS was similar in identifying DA functions in the first stage; however, surprisingly, TSWRS performed much better than FishTaco in identifying BR_p_ microbes in the second stage. LCM, WRS, ANCOM-BC, and LINDA performed worse than the two-stage methods at detecting BR_p_ microbes, as expected, because DA microbes not associated with functional shifts were treated as false positives. TSLC was the only method that controlled the false discovery rate (FDR) at 0.05. As the sample size increased, the merits of TSLC became more apparent: a much higher true positive rate (TPR) than FishTaco and TSWRS, while controlling the FDR at 0.05.

**Figure 2:**
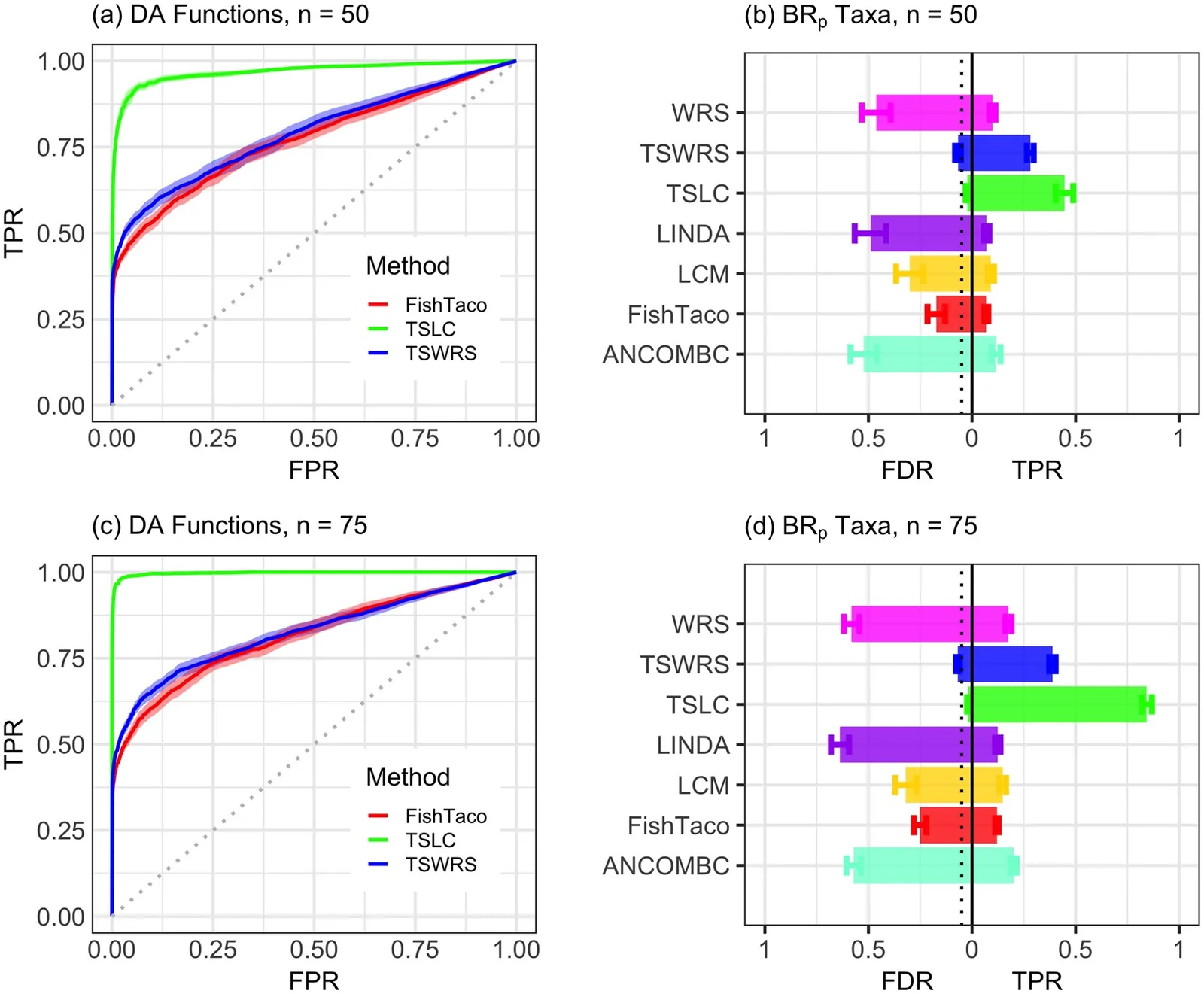
Simulation results for the negative binomial model from the first stage (a) and the second stage (b) with n=50 subjects per group, and the first stage (c) and the second stage (d) with n=75 subjects per group. The ROC plots on the left show the results of the first stage for each of the two-stage methods used to identify DA functions. The boxplots on the right show the TPR and the FDR for each method used to find BR_p_ microbes, including the typical one-stage method.

### 2.5 Microbial profiles by LN models

We considered the case in which the relative abundances of all microbes are jointly generated by a LN model, i.e. microbes are correlated with one another. Figure 3a shows performance comparison results for identifying DA functions in the first stage, and Fig. 3b shows performance comparison results for identifying BR_p_ microbes in the second stage when n=50. Figure 3c and d shows performance comparison results when n=75. Similar to the NB model setting, TSLC outperformed both FishTaco and TSWRS in identifying BR_p_ microbes. FishTaco and TSWRS performed similarly in identifying DA functions, but TSWRS performed better than FishTaco in identifying BR_p_ microbes. ANCOM-BC, LINDA, LCM, and WRS performed worse than the two-stage methods, as expected. TSLC was the only method that controlled the FDR at 0.05. Its TPR was also satisfactory when n=75, whereas the other methods had TPRs below 50%.

**Figure 3:**
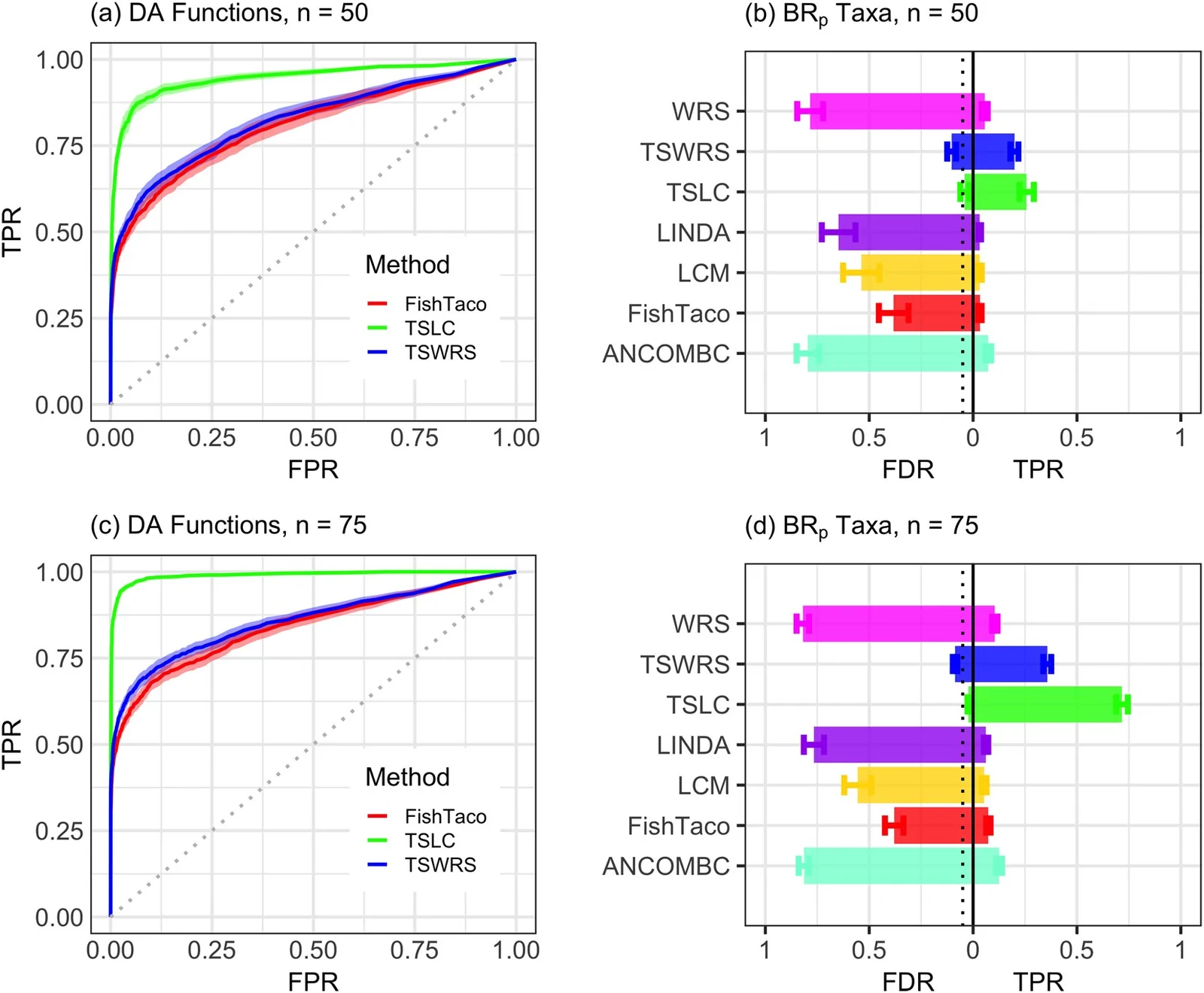
Simulation results for the logistic normal model from the first stage (a) and the second stage (b) with n=50 subjects per group, and the first stage (c) and the second stage (d) with n=75 subjects per group. The ROC plots on the left show the results of the first stage for each of the two-stage methods used to identify DA functions. The boxplots on the right show the TPR and the FDR for each method used to find BR_p_ microbes, including the typical one-stage method.

### 2.6 Permuted real microbiome data

All the methods considered in this simulation study controlled the false positive rate at a given threshold (FDR ≤0.05).

## 3 Real data analysis

### 3.1 Analysis on oral microbiome dataset

Dental caries is an infectious disease initiated by dental plaque formed by oral microorganisms and characterized by the demineralization of dental hard tissues, mediated by microbial metabolites within the plaque. Previous microbiome studies have shown that caries is associated with significant alterations in the microbiome. However, these studies focus only on taxonomic data and do not connect the microbiomes to the potential functions they perform, such as carbohydrate degradation and the generation of an acidic environment, which are critical for caries development (Pitts *et al.* 2017). To identify microbes related to metagenome-based functional differences in patients with dental caries (i.e. BR_p_ microbes), we used HUMAnN 3.0 to obtain a pathway profile and relevant microbes to each pathway in an oral microbiome dataset (OMEI dataset) consisting of patients with cavities (n=73) and healthy controls (n=42), which is a subset of the parent study (Yang *et al.* 2024). We filtered out low-abundant and low-frequent functions and microbes. Specifically, if the maximum relative abundance of a pathway (or a taxon) was smaller than 0.5% or it appeared in fewer than 20% of samples, it was removed from the metagenome-based functional and taxonomic profiles, leaving us with 346 metabolic pathways and 191 species. In the analysis, we adjusted for two covariates: age and smoking status.

The following 27 pathways were detected as DA pathways and had at least one known BR_p_ microbe associated with them: UMP biosynthesis II (denoted by P1 in Fig. 4), UMP biosynthesis III (P2), (5Z)-dodecenoate biosynthesis II (P3), petroselinate biosynthesis (P4), palmitate biosynthesis (P5), superpathway of unsaturated fatty acids biosynthesis (P6), superpathway of fatty acids biosynthesis (P7), L-ornithine biosynthesis II (P8), L-lysine biosynthesis I (P9), superpathway of L-methionine biosynthesis (P10), 2-carboxy-1,4-naphthoquinol biosynthesis (P11), superpathway of menaquinol-9 biosynthesis (P12), superpathway of menaquinol-6 biosynthesis (P13), superpathway of demethylmenaquinol-6 biosynthesis I (P14), superpathway of menaquinol-10 biosynthesis (P15), superpathway of heme b biosynthesis from glutamate (P16), CMP-legionaminate biosynthesis I (P17), methylerythritol phosphate pathway II (P18), pentose phosphate pathway (non-oxidative branch) II (P19), acetyl-CoA fermentation to butanoate II (P20), succinate fermentation to butanoate (P21), 4-aminobutanoate degradation V (P22), L-arginine degradation XIV (P23), 4-deoxy-L-threo-hex-4-enopyranuronate degradation (P24), starch degradation III (P25), adenosine nucleotides degradation II (P26), and chitin degradation II (P27). Additionally, we identified 16 DA pathways without any known DA species associated with them. These pathways are listed in the Supplementary File. In the second stage, we identified 35 BR_p_ microbes that were significantly associated with at least one pathway. *Streptococcus pneumoniae* was associated with pathways P4, P5, P6, P7, P10, P11, P16, and P18. *Streptococcus oralis* appeared in pathways P1, P2, P19, and P20. The other identified BR_p_ microbes were: *Streptococcus vestibularis* (P1, P2, P19); *Veillonella parvula* (P1, P2, P11); *Aggregatibacter aphrophilus* (P12, P13, P15); *Actinomyces oris* (P18, P19, P22); *Streptococcus salivarius* (P1, P19); *Haemophilus sp HMSC71H05* (P3, P11); *Actinomyces odontolyticus* (P9, P19); *Campylobacter concisus* (P16, P17); *Prevotella shahii* (P1); *Streptococcus milleri* (P1); *Granulicatella adiacens* (P4); *Pseudopropionibacterium propionicum* (P8); *Rothia mucilaginosa* (P9); *Haemophilus parainfluenzae* (P10); *Veillonella atypica* (P11); *Veillonella sp T110116* (P11); *Aggregatibacter sp oral taxon 458* (P14); *Veillonella dispar* (P16); *Fusobacterium periodonticum* (P17); *Actinomyces sp oral taxon 170* (P18); *Actinomyces viscosus* (P18); *Corynebacterium durum* (P18); *Cutibacterium acnes* (P18); *Tannerella forsythia* (P19); *Fusobacterium nucleatum* (P20); *Streptococcus mitis* (P20); *Bacteroidetes oral taxon 274* (P21); *Eubacterium nodatum* (P23); *Treponema lecithinolyticum* (P24); *Actinomyces sp oral taxon 448* (P25); *Atopobium parvulum* (P26); *Streptococcus gordonii* (P27); *Streptococcus sanguinis* (P27), which are shown in Fig. 4.

**Figure 4:**
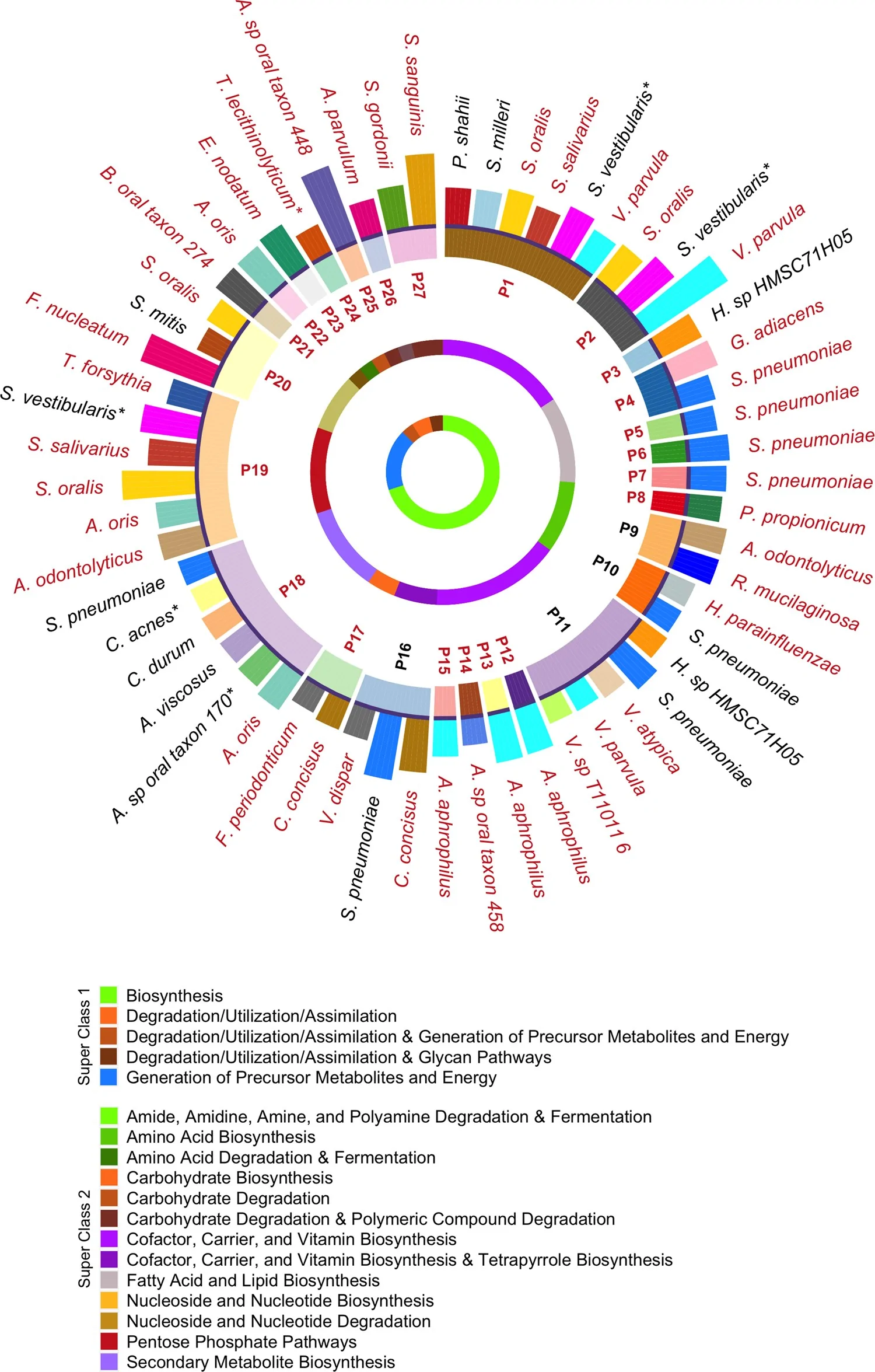
DA pathways and BR_p_ microbes for the OMEI dataset. Each bar represents the estimated coefficient for a pathway or a microbe, with the caries-free group as the reference. Red labels indicate a positive coefficient and black labels indicate a negative coefficient. The colors of the inner rings denote the superclasses of the pathways. The asterisk on the microbe name indicates that it was identified by both the one-stage and two-stage approaches.

L-lysine biosynthesis I (P9), superpathway of L-methionine biosynthesis (P10), 2-carboxy-1,4-naphthoquinol biosynthesis (P11), and superpathway of heme b biosynthesis from glutamate (P16) were significantly more abundant in caries-free individuals. These pathways support microbial communities by enhancing amino acid synthesis and cofactor production, which are necessary for oxidative metabolism and redox homeostasis, traits generally associated with oral microbial stability and resilience to acid stress (Noor Al-Hebshi *et al.* 2018).

In contrast, the other 23 pathways were enriched in the caries group and involved in nucleotide biosynthesis (P1, P2), fatty acid production (P3–P7), acidogenic fermentation (P20–P23), and complex carbohydrate degradation (P24–P27). Notably, *V. parvula*, a species frequently associated with cariogenic biofilms, was identified as a major contributor to UMP biosynthesis II and III (P1, P2), indicating its role in supporting nucleotide production and microbial proliferation in cariogenic environments. In addition, *E. nodatum*, a species linked to dysbiotic and inflammatory conditions, contributed to L-arginine degradation XIV (P23). While arginine metabolism can be protective in oral health by generating ammonia to buffer pH, the degradation route represented by L-arginine degradation XIV may reduce this buffering capacity and instead support metabolic pathways that favor acidogenic and proteolytic activity within caries-associated communities (Nascimento *et al.* 2019).

We also applied LCM to a taxonomic profile of the OMEI dataset obtained by MetaPhlAn 3.0 (Beghini *et al.* 2021). The identified set of DA microbes by this one-stage approach was very different from the set of BR_p_ microbes from the two-stage approach: *Actinomyces massiliensis*; *Actinomyces sp oral taxon 170*; *Bifidobacterium dentium*; *C. acnes*; *Prevotella multiformis*; *Prevotella oulorum*; *Prevotella pleuritidis*; *Streptococcus sp M334*; *S. vestibularis*; *Mogibacterium diversum*; *Lachnospiraceae bacterium oral taxon 096*; *Eggerthia catenaformis*; *Dialister invisus*; *Leptotrichia sp oral taxon 215*; *Neisseria sp oral taxon 014*; *Treponema denticola*; *T. lecithinolyticum*. Only *A. sp oral taxon 170*, *C. acnes*, *S. vestibularis*, and *T. lecithinolyticum* appeared in both the one-stage (LCM) and two-stage (TSLC) approaches.

### 3.2 Analysis on IBD dataset

Ulcerative colitis (UC) is a chronic inflammatory bowel disease (IBD) of the colon that can result in continuous colonic inflammation, erosions, bleeding, and ulcerations (Gajendran *et al.* 2019, Steube *et al.* 2023). In addition to genetic and environmental factors predisposing to IBD, structural alterations in the gut microbiome have been associated with UC (Norman *et al.* 2015, Gajendran *et al.* 2019). To identify microbes associated with functional shifts in metagenome-based functions in patients with UC, we analysed the IBD dataset of Franzosa *et al.* (2019) using TSLC. We downloaded the whole-metagenome sequencing data for patients with UC (n=53) and healthy controls (n=34) from the NCBI Sequence Read Archive under BioProject PRJNA400072. Using strategies similar to those used in the analysis of the OMEI dataset, we identified 172 metabolic pathways and 156 species. In the analysis, we adjusted for five covariates (one continuous and four binary): age, antibiotic use, immunosuppressant use, mesalamine use, and steroid use.

We found 17 DA pathways, and 12 of them had at least one known BR_p_ microbe associated with it: homolactic fermentation (denoted by P1 in Fig. 5); glycolysis I (P2); glycolysis II (P3); pentose phosphate pathway (P4); L-lysine biosynthesis I (P5); superpathway of L-tryptophan biosynthesis (P6); L-tryptophan biosynthesis (P7); superpathway of coenzyme A biosynthesis I (P8); thiamin phosphate formation from pyrithiamine and oxythiamine (P9); flavin biosynthesis I (P10); superpathway of thiamin diphosphate biosynthesis I (P11); superpathway of glucose and xylose degradation (P12). The other five DA pathways that did not have any known DA species associated with them are listed in the Supplementary File.

**Figure 5:**
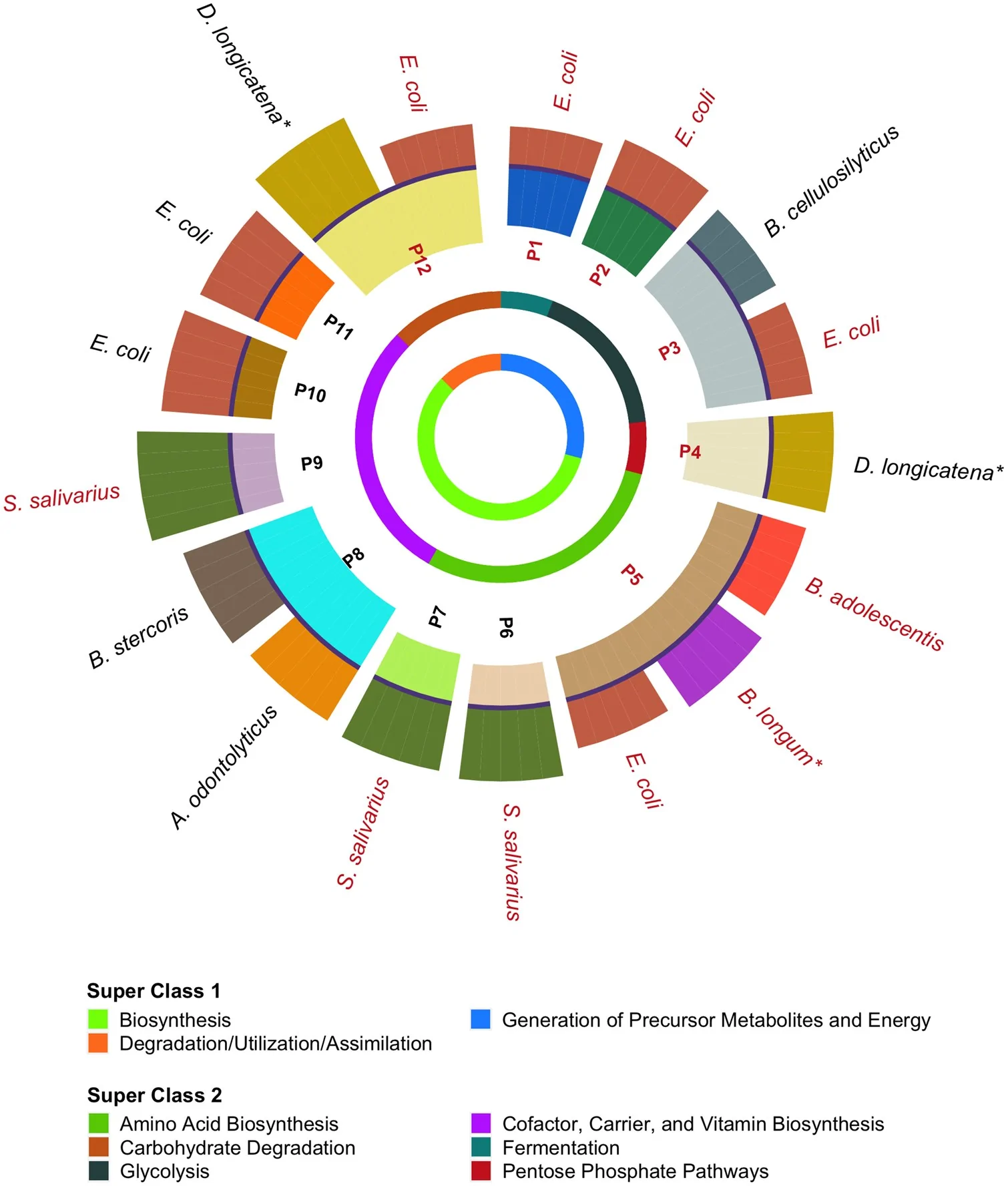
DA pathways and BR_p_ microbes for the IBD dataset. Each bar represents the estimated coefficient for a pathway or a microbe, with the control group as the reference. Red labels indicate a positive coefficient and black labels indicate a negative coefficient. The colors of the inner rings denote the superclasses of the pathways. The asterisk on the microbe name indicates that it was identified by both the one-stage and two-stage approaches.

In the second stage, we identified eight BR_p_ microbes that were significantly associated with pathways. *Escherichia coli* was associated with pathways P1, P2, P3, P5, P10, P11, and P12. *S. salivarius* was associated with pathways P6, P7, and P9. The other identified BR_p_ microbes were: *Dorea longicatena* (P4, P12); *Bacteroides cellulosilyticus* (P3); *Bifidobacterium adolescentis* (P5); *Bifidobacterium longum* (P5); *A. odontolyticus* (P8); *Bacteroides stercoris* (P8), which are shown in Fig. 5.

Franzosa et al. (2019) performed untargeted metabolomic profiling of these same samples. Amongst the metabolites enriched in controls were flavonoids, consistent with our results showing increased flavin biosynthesis pathway in controls. More recently, Valdés-Mas et al. (2025), using a metagenome-informed metaproteomics analysis of a German UC cohort compared to age-matched healthy controls, also found increased glycolysis pathway metabolites in participants with UC, as well as increased microbial pentose phosphate pathway metabolites in *E. coli* in UC. *E. coli*, particularly pathogenic strains, has been known to play a role in the chronic inflammation in UC, with multiple mechanisms proposed (Iyer *et al.* 2018, Mirsepasi-Lauridsen *et al.* 2019). A randomized trial comparing nonpathogenic *E. coli* administration with mesalamine for UC showed equivalent outcomes in maintaining remission (Rembacken *et al.* 1999), further demonstrating that *E. coli* is a key player in chronic inflammation in UC. This highlights the importance of considering functional redundancy in studies to identify biologically relevant microbes prior to further functional studies and therapeutic applications.

When applying LCM to a taxonomic profile of the IBD dataset obtained using MetaPhlAn 3.0, we identified 28 DA microbes: *Methanobrevibacter smithii*; *Actinomyces sp HMSC035G02*; *B. longum*; *Gordonibacter pamelaeae*; *Bacteroides fragilis*; *Prevotella copri*; *Alistipes obesi*; *Lactococcus lactis*; *Clostridium sp CAG 58*; *Clostridium sp CAG 75*; *Eubacterium eligens*; *Eubacterium ramulus*; *Blautia sp AF19 10LB*; *Coprococcus comes*; *D. longicatena*; *Dorea sp CAG 317*; *Roseburia intestinalis*; *Eubacterium siraeum*; *Ruminococcaceae bacterium D16*; *Ruminococcus bicirculans*; *Ruminococcus bromii*; *Subdoligranulum sp*; *Clostridium innocuum*; *Firmicutes bacterium CAG 94*; *Firmicutes bacterium CAG 103*; *H. parainfluenzae*; *V. dispar*; *Proteobacteria bacterium CAG 139*. Among these 28 DA microbes, only *D. longicatena* and *B. longum* appeared in the result from the proposed method.

## 4 Discussion

Identifying microbes associated with phenotypic changes is critical, particularly for therapeutic applications. However, DA analysis is typically performed using taxonomic profiles, which neglects functional redundancy, gene copy number variation, and the contribution of unknown microbes, thereby leading to the identification of microbes not associated with phenotypic differences. To address these issues, we proposed a two-stage method (TSLC) that uses $\ell_{1}$-penalized log-contrast regression models and debiasing procedures. We demonstrated its outperformance in identifying biologically relevant microbes in simulation studies.

In simulation studies, the gene copy number was set to 1 for all functions in all microbes to enable a direct comparison between the one-stage and two-stage approaches. In this setting, microbes identified by the two-stage approach were expected to be a subset of those identified by the one-stage approach, since some microbes are not relevant to functional shifts. Random variation in gene copy numbers does not affect TSLC performance, as TSLC requires only the product of microbial abundance and their gene copy numbers.

In the real data analysis, the set of microbes identified by the two-stage approach was distinct from that by the one-stage approach, highlighting the consequence of disregarding functional redundancy, gene copy number variation, and the contribution of unknown microbes. Therefore, the two-stage approach is essential for determining biologically relevant microbes that could serve as targets for therapeutic applications. The BR_p_ microbes identified by TSLC in this study are metagenome-based functions rather than differentially expressed (DE) functions. However, microbes associated with DE functions can be identified by TSLC if both metatranscriptomic and metagenomic data are available, as HUMAnN can take metatranscriptomic and metagenomic data as inputs and produce a metatranscriptome-based functional profile and the contributions of relevant microbes to each function.

Although the outputs from HUMAnN were utilized in the real data examples, the proposed method is applicable to any profiling methods as long as they provide a functional profile *F* and the contribution of taxa to functional shifts $M_{\left(j\right)}$ for all *j*, alternatively *M* and *G*, separately. The current version of the proposed method is limited to cross-sectional data with two groups. Future work will focus on extending the proposed method to accommodate multi-group (i.e. >2 groups) and longitudinal outcomes.

## Supplementary Material

vbag297_Supplementary_Data
