# Supplementary material for: TSLC: differential abundance analysis method for identifying biologically relevant known microbes
Source: Bioinform Adv. 2026 Oct 6;6(1):vbag297. doi: 10.1093/bioadv/vbag297 (PMC13674353; doi:10.1093/bioadv/vbag297)
Supplement: Supplementary file 1 [file vbag297_supplementary_data.pdf]

TSLC: Differential abundance analysis method for identifying biologically relevant known microbes

Supplementary Materials

By Robert Beblavy, Cynthia L. Monaco, Cassandra E. Newkirk, Jin Xiao, and Michael B. Sohn

A Algebraic Operators in Simplex Space

For two compositions of k-components, $\boldsymbol{\eta}$ and $\boldsymbol{\zeta}$, in a $k-1$ simplex space, $\mathbb{S}^{k-1}$, the perturbation operator is defined as

$$\boldsymbol{\eta} \oplus \boldsymbol{\zeta} = \left(\frac{\eta_1 \zeta_1}{\sum_{j=1}^k \eta_j \zeta_j}, \frac{\eta_2 \zeta_2}{\sum_{j=1}^k \eta_j \zeta_j}, \dots, \frac{\eta_k \zeta_k}{\sum_{j=1}^k \eta_j \zeta_j} \right)^\top,$$

and the power transformation for a composition $\boldsymbol{\eta}$ by a scalar υ as

$$\boldsymbol{\eta}^\upsilon = \left(\frac{\eta_1^\upsilon}{\sum_{j=1}^k \eta_j^\upsilon}, \frac{\eta_2^\upsilon}{\sum_{j=1}^k \eta_j^\upsilon}, \dots, \frac{\eta_k^\upsilon}{\sum_{j=1}^k \eta_j^\upsilon} \right)^\top.$$

B Estimation of Parameters

Let $q(y_i \boldsymbol{x}_{i\cdot}^\top \boldsymbol{\alpha}) = -\mathcal{L}(y_i | \log(\boldsymbol{f}_{i\cdot}), \boldsymbol{\alpha}) = -\log \Phi(y_i \boldsymbol{x}_{i\cdot}^\top \boldsymbol{\alpha})$. Using the Taylor expansion, it can be given by

$$\begin{aligned} q(y_i \boldsymbol{x}_{i\cdot}^\top \boldsymbol{\alpha}) &= q(y_i \boldsymbol{x}_{i\cdot}^\top \underline{\boldsymbol{\alpha}}) + (\boldsymbol{\alpha} - \underline{\boldsymbol{\alpha}})^\top G(y_i \boldsymbol{x}_{i\cdot}^\top \underline{\boldsymbol{\alpha}}) + \frac{1}{2} (\boldsymbol{\alpha} - \underline{\boldsymbol{\alpha}})^\top H(y_i \boldsymbol{x}_{i\cdot}^\top \tilde{\boldsymbol{\alpha}})(\boldsymbol{\alpha} - \underline{\boldsymbol{\alpha}}) \\ &= \frac{1}{2} \boldsymbol{\alpha}^\top H(y_i \boldsymbol{x}_{i\cdot}^\top \tilde{\boldsymbol{\alpha}}) \boldsymbol{\alpha} + G(y_i \boldsymbol{x}_{i\cdot}^\top \underline{\boldsymbol{\alpha}})^\top \boldsymbol{\alpha} - \underline{\boldsymbol{\alpha}}^\top H(y_i \boldsymbol{x}_{i\cdot}^\top \tilde{\boldsymbol{\alpha}})^\top \boldsymbol{\alpha} + C, \end{aligned}$$

where $G(y_i \boldsymbol{x}_{i\cdot}^\top \underline{\boldsymbol{\alpha}}) = \nabla_\alpha q(y_i \boldsymbol{x}_{i\cdot}^\top \underline{\boldsymbol{\alpha}})$, $H(y_i \boldsymbol{x}_{i\cdot}^\top \tilde{\boldsymbol{\alpha}}) = \nabla_\alpha^2 q(y_i \boldsymbol{x}_{i\cdot}^\top \tilde{\boldsymbol{\alpha}})$, $\tilde{\boldsymbol{\alpha}}$ a vector that lies between $\underline{\boldsymbol{\alpha}}$ and $\boldsymbol{\alpha}$, and C is a constant with respect to $\boldsymbol{\alpha}$. Since $\sum_{i=1}^n H(y_i \boldsymbol{x}_{i\cdot}^\top \tilde{\boldsymbol{\alpha}}) \succeq 0$, finding a solution minimizing

$\sum_{i=1}^{n} q(y_i \boldsymbol{x}_{i\cdot}^\top \boldsymbol{\alpha})$ is equivalent to finding a solution of $\nabla_\alpha q(y_i \boldsymbol{x}_{i\cdot}^\top \boldsymbol{\alpha}) = 0$, that is,

$$\hat{\boldsymbol{\alpha}} = \underset{\boldsymbol{\alpha}}{\operatorname{argmin}} \left\{ \frac{1}{n} \sum_{i=1}^{n} q(y_i \boldsymbol{x}_{i\cdot}^\top \boldsymbol{\alpha}) \right\}$$

$$\Updownarrow$$

$$\hat{\boldsymbol{\alpha}} = \underline{\boldsymbol{\alpha}} - \left(\sum_{i=1}^{n} H(y_i \boldsymbol{x}_{i\cdot}^\top \tilde{\boldsymbol{\alpha}}) \right)^{-} \left(\sum_{i=1}^{n} G(y_i \boldsymbol{x}_{i\cdot}^\top \underline{\boldsymbol{\alpha}}) \right),$$

where A^- is the Moore-Penrose inverse of a matrix A. Note that $\nabla_\alpha q(y_i \boldsymbol{x}_{i\cdot}^\top \boldsymbol{\alpha}) = -\xi_i(\boldsymbol{\alpha}) \boldsymbol{x}_{i\cdot}$, where $\xi_i(\boldsymbol{\alpha}) = y_i \phi(y_i \boldsymbol{x}_{i\cdot}^\top \boldsymbol{\alpha}) / \Phi(y_i \boldsymbol{x}_{i\cdot}^\top \boldsymbol{\alpha})$ and $\nabla_\alpha^2 q(y_i \boldsymbol{x}_{i\cdot}^\top \boldsymbol{\alpha}) = \xi_i(\boldsymbol{\alpha})[\boldsymbol{x}_{i\cdot}^\top \boldsymbol{\alpha} + \xi_i(\boldsymbol{\alpha})] \boldsymbol{x}_{i\cdot} \boldsymbol{x}_{i\cdot}^\top$. Substituting these terms, we have

$$\hat{\boldsymbol{\alpha}} = \underline{\boldsymbol{\alpha}} + (X^\top D X)^- X^\top \boldsymbol{\xi}(\underline{\boldsymbol{\alpha}}) = (X^\top D X)^- X^\top D \boldsymbol{u},$$

where D is an $n \times n$ diagonal matrix with the i-th diagonal term $d_i = \xi_i(\tilde{\boldsymbol{\alpha}})[\boldsymbol{x}_{i\cdot}^\top \tilde{\boldsymbol{\alpha}} + \xi_i(\tilde{\boldsymbol{\alpha}})]$, $\boldsymbol{\xi}(\underline{\boldsymbol{\alpha}}) = (\xi_1(\underline{\boldsymbol{\alpha}}), \ldots, \xi_n(\underline{\boldsymbol{\alpha}}))^\top$, and $\boldsymbol{u} = X \underline{\boldsymbol{\alpha}} + D^- \boldsymbol{\xi}(\underline{\boldsymbol{\alpha}})$. This is, given $\tilde{\boldsymbol{\alpha}}$ and $\underline{\boldsymbol{\alpha}}$, the solution of a weighted least squares problem with a weight matrix D, a dependent variable $\boldsymbol{u}$, and independent variables X, that is,

$$\hat{\boldsymbol{\alpha}} = \underset{\boldsymbol{\alpha}}{\operatorname{argmin}} \| D^{1/2}(\boldsymbol{u} - X\boldsymbol{\alpha}) \|_2^2.$$

Therefore, the optimization problem (2) can be expressed as

$$\hat{\boldsymbol{\alpha}} = \underset{\boldsymbol{\alpha}}{\operatorname{argmin}} \left\{ \frac{1}{2n} \| D^{1/2}(\boldsymbol{u} - X\boldsymbol{\alpha}) \|_2^2 + \lambda \sum_{j=1}^{p} |\alpha_j| \right\},$$
$$\text{subject to } \sum_{j=1}^{p} \alpha_j = 0,$$

The Augmented Lagrangian for this optimization problem is given by,

$$L = \frac{1}{2n} \left\| D^{1/2}(\boldsymbol{u} - X\boldsymbol{\alpha}) \right\|_2^2 + \lambda \sum_{j=1}^{p} |\alpha_j| + \frac{\omega}{2} \left(\sum_{j=1}^{p} \alpha_j + \eta \right)^2,$$

where η is a scaled Lagrange multiplier and $\omega > 0$ is a penalty parameter. Now observe that,

$$\begin{aligned} \frac{1}{2n} \left\| D^{1/2}(\boldsymbol{u} - X\boldsymbol{\alpha}) \right\|_2^2 &= \frac{1}{2n} (\boldsymbol{u} - X\boldsymbol{\alpha})^T D (\boldsymbol{u} - X\boldsymbol{\alpha}) \\ &= \frac{1}{2n} \left(\boldsymbol{u}^T D \boldsymbol{u} - 2\boldsymbol{\alpha}^T X^T D \boldsymbol{u} + \boldsymbol{\alpha}^T X^T D X \boldsymbol{\alpha} \right) \end{aligned}$$

Taking the gradient of this quantity with respect to $\boldsymbol{\alpha}$ yields,

$$\nabla_{\boldsymbol{\alpha}} \left(\frac{1}{2n} \left\| D^{1/2}(\boldsymbol{u} - X\boldsymbol{\alpha}) \right\|_2^2 \right) = -\frac{1}{n} X^T D \left(\boldsymbol{u} - X\boldsymbol{\alpha} \right)$$

We can write each component of the gradient as follows,

$$\frac{\partial}{\partial \alpha_j}\left(\frac{1}{2n}\left\|D^{1/2}(\boldsymbol{u}-X\boldsymbol{\alpha})\right\|_2^2\right) = -\frac{1}{n}\boldsymbol{x}_{\cdot j}^T D\left(\boldsymbol{u}-X\boldsymbol{\alpha}\right)$$
$$= -\frac{1}{n}\boldsymbol{x}_{\cdot j}^T D\left(\boldsymbol{u}-X_{-j}\boldsymbol{\alpha}_{-j}-\boldsymbol{x}_{\cdot j}\alpha_j\right)$$

Using this, we can express the derivative of the Augmented Lagrangian with respect to α_j as follows,

$$\frac{\partial L}{\partial \alpha_j} = -\frac{1}{n}\boldsymbol{x}_{\cdot j}^T D\left(\boldsymbol{u}-X_{-j}\boldsymbol{\alpha}_{-j}-\boldsymbol{x}_{\cdot j}\alpha_j\right)+\lambda\partial_{|\alpha_j|}+\omega\left(\alpha_j+\sum_{l\neq j}\alpha_l+\eta\right)$$
$$= -\frac{1}{n}\boldsymbol{x}_{\cdot j}^T D\left(\boldsymbol{u}-X_{-j}\boldsymbol{\alpha}_{-j}\right)+\frac{1}{n}\boldsymbol{x}_{\cdot j}^T D\boldsymbol{x}_{\cdot j}\alpha_j+\lambda\partial_{|\alpha_j|}+\omega\alpha_j+\omega\left(\sum_{l\neq j}\alpha_l+\eta\right)$$

Now setting $\frac{\partial L}{\partial \alpha_j}=0$ allows us to find the parameter estimates at iteration k. To do so, we can solve,

$$\hat{\alpha}_j^{(k)} = \frac{1}{m_j}S_\lambda\big\{h(X,\boldsymbol{\alpha}_{-j})-\omega g(\boldsymbol{\alpha}_{-j},\eta)\big\},$$

where $m_j = \|\boldsymbol{x}_{\cdot j}\|_2^2/n+\omega/p$, $\boldsymbol{x}_{\cdot j}$ is the j-th column vector of X, $S_\lambda(t)=\text{sign}(t)(|t|-\lambda)_+$ is the soft thresholding operator,

$$h(X,\boldsymbol{\alpha}_{-j}) = \frac{1}{n}\boldsymbol{x}_{\cdot j}^\top D^{(k)}\big(\boldsymbol{u}^{(k)}-X_{-j}\boldsymbol{\alpha}_{-j}\big),$$
$$g(\boldsymbol{\alpha}_{-j},\eta) = \frac{1}{p}\sum_{\ell\neq j}\alpha_\ell^{(k+1)}+\frac{1}{\sqrt{p}}\eta^{(k)},$$

C De-biasing Procedure

Let $\widehat{\Sigma} = X^\top\widehat{D}X/n$, where $\widehat{D}$ is an estimate of D obtained from Algorithm 1, $\boldsymbol{e}_j\in\mathbb{R}^p$ be the vector with one at the j-th position and zero everywhere else, and γ be some constant. The matrix $\widetilde{\Theta}$ can be obtained from Algorithm S1.

Algorithm S1 Constructing a de-biased estimator

1: **for** $j=1$ to p **do**
2: $\quad\hat{\boldsymbol{\theta}}_j \leftarrow \min_\theta \boldsymbol{\theta}^\top\widehat{\Sigma}\boldsymbol{\theta}\quad$ s.t. $\quad\|\widehat{\Sigma}\boldsymbol{\theta}-(I_p-J_p/p)\boldsymbol{e}_j\|_\infty\leq\gamma$
3: **end for**
4: $\widehat{\Theta}\leftarrow\big(\hat{\boldsymbol{\theta}}_i,\ldots,\hat{\boldsymbol{\theta}}_p\big)^\top;\qquad\widetilde{\Theta}\leftarrow(I_p-J_p/p)\widehat{\Theta}$
5: $\hat{\boldsymbol{\alpha}}^{de}\leftarrow\hat{\boldsymbol{\alpha}}+\frac{1}{n}\widetilde{\Theta}X^\top\widehat{D}\left(\hat{\boldsymbol{u}}-X\hat{\boldsymbol{\alpha}}\right)$

D DA Pathways for OMEI Data

The following pathways were detected as DA, but did not have any associated DA species: Pyrimidine Deoxyribonucleotides de novo Biosynthesis III; Superpathway of Allantoin Degradation in Yeast; Pyruvate Fermentation to Butanoate; Superpathway of Histidine, Purine, and Pyrimidine Biosynthesis; Palmitate Biosynthesis; CMP-N-Acetylneuraminate Biosynthesis I; Purine Nucleotides Degradation II; Pyruvate Fermentation to Butanol I; Pyruvate Fermentation to Acetone; Superpathway of Clostridium Acetobutylicum Acidogenic Fermentation; Superpathway of Clostridium Acetobutylicum Solventogenic Fermentation; Superpathway of Guanosine Nucleotides Degradation; Pyruvate Fermentation to Butanol II; Superpathway of Thiamine Diphosphate Biosynthesis II; Superpathway of Pyrimidine Deoxyribonucleotides de novo Biosynthesis; Oleate Biosynthesis IV.

E DA Pathways for IBD Data

The following pathways were detected as DA, but did not have any associated DA species: Superpathway of Adenosylcobalamin Salvage from Cobinamide I; Methanogenesis from Acetate; Phosphopantothenate Biosynthesis I; Rubisco Shunt; Starch Biosynthesis.
